# Supplementary material for: A cognitive neurogenetic approach to uncovering the structure of executive functions
Source: Nat Commun. 2022 Aug 6;13:4588. doi: 10.1038/s41467-022-32383-0 (PMC9357028; doi:10.1038/s41467-022-32383-0)
Supplement: Supplementary file 1 — Supplementary Information [file 41467_2022_32383_MOESM1_ESM.pdf]

A cognitive neurogenetic approach to uncovering the structure of executive functions

Supplementary Information

This file contains: Supplementary results, 3 supplementary figures and 8 supplementary tables.

Supplementary results

Functional enrichment of genes that showed enhanced expression in MFG and SCG

We used Toppgene suite to perform functional annotation of candidate genes[1] (https://toppgene.cchmc.org/). It detects functional enrichment of input gene list based on Go Term, Mouse phenotype, Disease, Pathway, Transcription factors and so on. The suite included multiple data sources, such as ***Gene Ontology (GO)***[2] (http://geneontology.org/), which contained GO Molecular Function, GO Biological Process and GO cellular component. ***Mouse phenotype*** (http://www.informatics.jax.org/). ***Pathway annotations*** included data from the Kyoto Encyclopedia of Genes and Genomes (KEGG)[3] (https://www.genome.jp/kegg/pathway.html), BioCarta (http://www.biocarta.com/genes/), BioCyc[4] (https://biocyc.org/), Reactome[5] (https://reactome.org/), GenMAPP[6], and Molecular Signature Database (MSigDb) (https://www.gsea-msigdb.org/gsea/msigdb/index.jsp). Human Phenotype Ontology (https://hpo.jax.org/app/), which included gene annotations to hereditary diseases. Hypergeometric distribution is used as the standard method to test statistical significance. The FDR[7] method was used for multiple comparisons correction.

MFG-related genes showed significant enrichment (see Supplementary Data). In terms of **GO molecular functions**, these genes were enriched for gated channel activity (p = 3.33 x 10^{-21}, FDR-BH corrected, hereafter), ion channel activity (p = 2.26 x 10^{-19}),

voltage-gated cation channel activity (p = 2.26 x 10^{-19}), cation channel activity (p = 4.44 x 10^{-19}), channel activity (p = 8.09 x 10^{-19}). In terms of **GO biological process**, these genes showed overrepresentation in synaptic signaling (p = 2.70 x 10^{-53}), anterograde trans-synaptic signaling (p = 2.70 x 10^{-53}), chemical synaptic transmission (p = 2.70 x 10^{-53}), trans-synaptic signaling (p = 9.04 x 10^{-53}) and cell-cell signaling (p = 3.62 x 10^{-41}). In terms of **GO cellular component**, the MFG-related genes are mainly enriched for synapse (p = 8.54 x 10^{-52}), neuron projection (p = 3.49 x 10^{-40}), glutamatergic synapse (p = 9.86 x 10^{-35}), synaptic membrane (p = 1.67 x 10^{-34}), and somatodendritic compartment (p = 1.21 x 10^{-33}). For **mouse phenotype,** these genes were enriched for abnormal synaptic transmission (p = 1.60 x 10^{-18}), abnormal CNS synaptic transmission (p = 1.59 x 10^{-16}), abnormal nervous system physiology (p = 1.81 x 10^{-16}), abnormal learning/memory/conditioning (p = 7.39 x 10^{-16}), abnormal cognition (p = 7.39 x 10^{-16}). In annotation of **pathway**, these genes showed enrichment in neuroactive ligand-receptor interaction (p = 6.49 x 10^{-14}), calcium signaling pathway (p = 1.05 x 10^{-12}), monoamine GPCRs (p = 1.78 x 10^{-8}), Heterotrimeric G-protein signaling pathway-Gi alpha and Gs alpha mediated pathway (p = 1.47 x 10^{-7}), and axon guidance (p = 3.06 x 10^{-7}). For **disease**, these genes are enriched in schizophrenia (p = 1.18 x 10^{-26}), chronic alcoholic intoxication (p = 1.43 x 10^{-15}), bipolar disorder (p = 1.43 x 10^{-15}), autistic disorders (p = 1.80 x 10^{-13}), mental disorders (p = 3.04 x 10^{-13}).

SCG-related genes also showed significant enrichment (see Supplementary Data). For **GO molecular functions:** signaling receptor binding (p = 1.97 x 10^{-9}, FDR-BH corrected, hereafter), channel activity (p = 2.36 x 10^{-7}), passive transmembrane transporter activity (p = 2.36 x 10^{-7}), metal ion transmembrane transporter activity (p = 6.32 x 10^{-7}), potassium channel activity (p = 6.32 x 10^{-7}). For **GO biological process:** synaptic signaling (p = 3.85 x 10^{-28}), trans-synaptic signaling (p = 1.09 x 10^{-27}), cell-cell signaling (p = 1.67 x 10^{-27}), anterograde trans-synaptic signaling (p = 2.24 x 10^{-27}), and chemical synaptic transmission (p = 2.24 x 10^{-27}). For **GO cellular component**: synapse (p = 7.85 x 10^{-35}), neuron projection (p = 1.90 x 10^{-31}), somatodendritic compartment (p = 2.28 x

10^{-25}), synaptic membrane (p = 4.12 x 10^{-20}), and dendritic tree (p = 4.12 x 10^{-20}). For **mouse phenotype**: abnormal nervous system physiology (p = 1.30 x 10^{-13}), abnormal CNS synaptic transmission (p = 1.79 x 10^{-10}), abnormal synaptic transmission (p = 4.67 x 10^{-10}), abnormal learning/memory/conditioning (p = 3.39 x 10^{-9}), abnormal cognition (p = 3.39 x 10^{-9}). For **pathway:** neuroactive ligand-receptor interaction (p = 1.44 x 10^{-5}), spinal cord injury (p = 3.58 x 10^{-5}), ensemble of genes encoding core extracellular matrix including ECM glycoproteins, collagens and proteoglycans (p = 5.96 x 10^{-5}), Myometrial relaxation and contraction pathways (p = 3.43 x 10^{-4}), and calcium regulation in the cardiac cell (p = 4.47 x 10^{-4}). For **disease:** anxiety (p = 7.79 x 10^{-13}), anxiety disorders (p = 1.30 x 10^{-12}), schizophrenia (p = 8.48 x 10^{-11}), mental disorders (p = 1.61 x 10^{-9}), bipolar disorder (p = 1.61 x 10^{-9}).

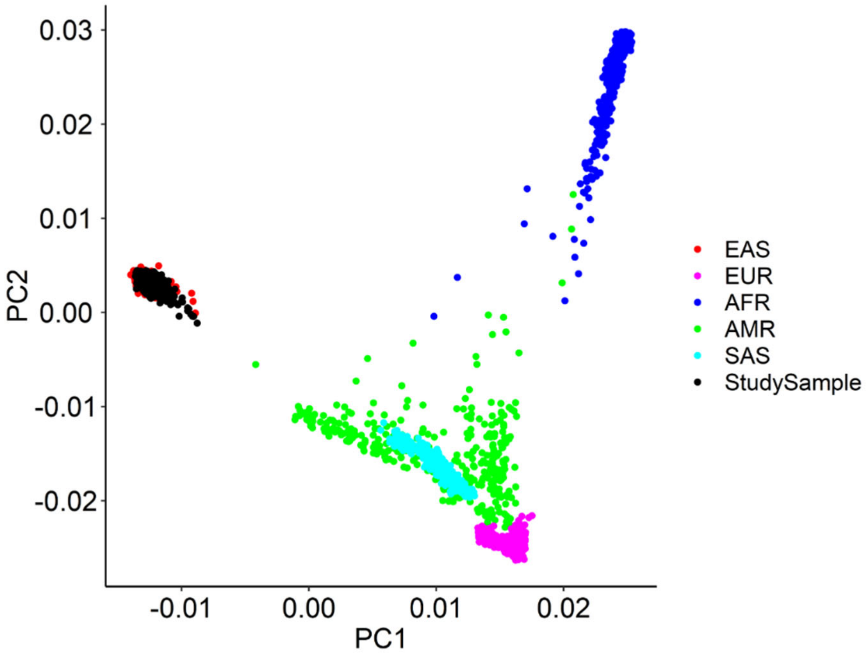

Supplementary Fig. 1. Principal component analysis (PCA) of 2110 subjects. The PCA is based on ~3 million common SNPs on 1000Genome phase3 data. EAS: East Asian, EUR: European, AFR: African, AMR: Ad Mixed American, SAS: South Asian, StudySample: 2110 subjects used in the current study. Source data are provided as a Source Data file.

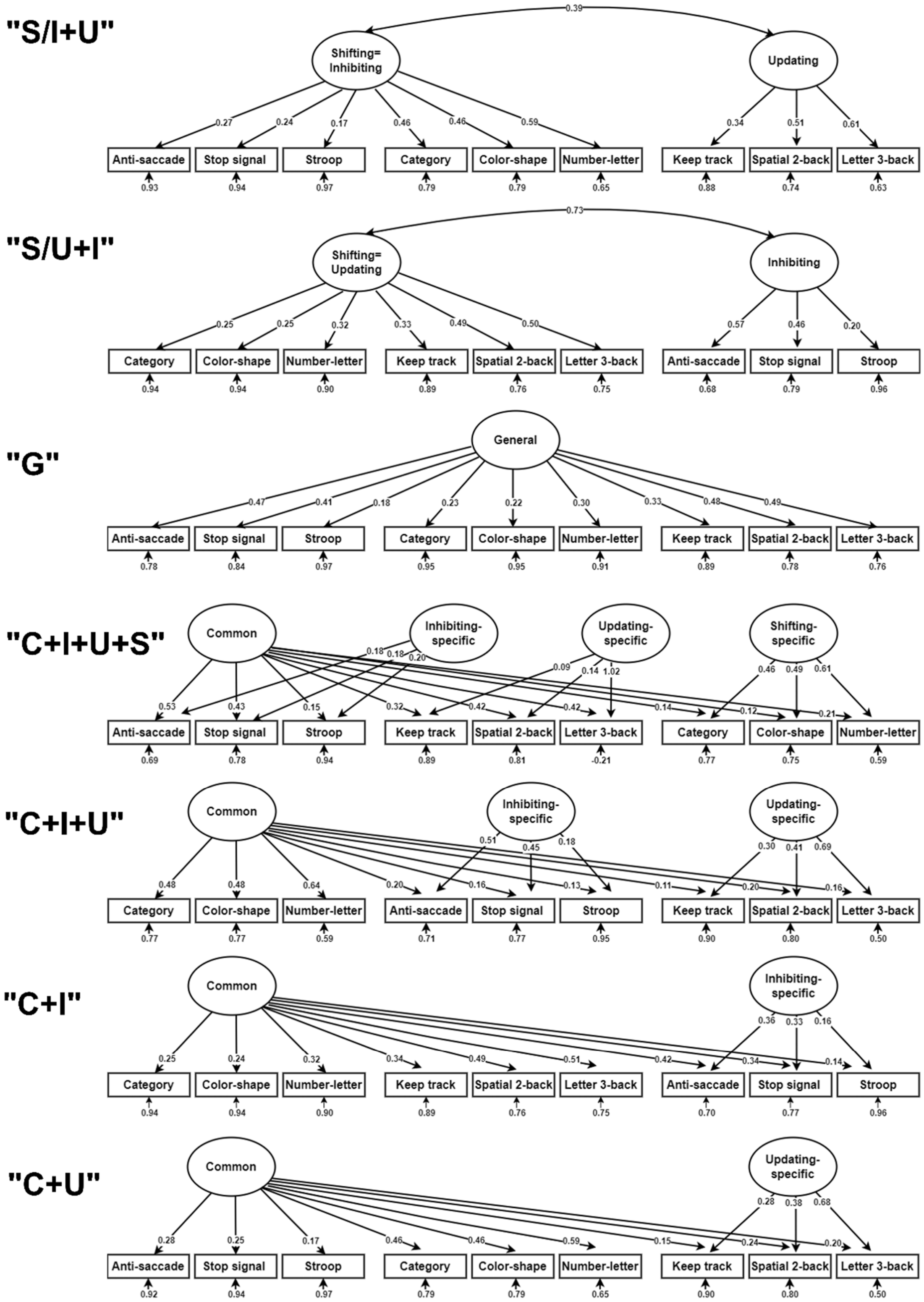

Supplementary Fig. 2. Latent variable models of EFs (seven other models). See Table 1 for fit statistics for these models. Source data are provided as a Source Data file.

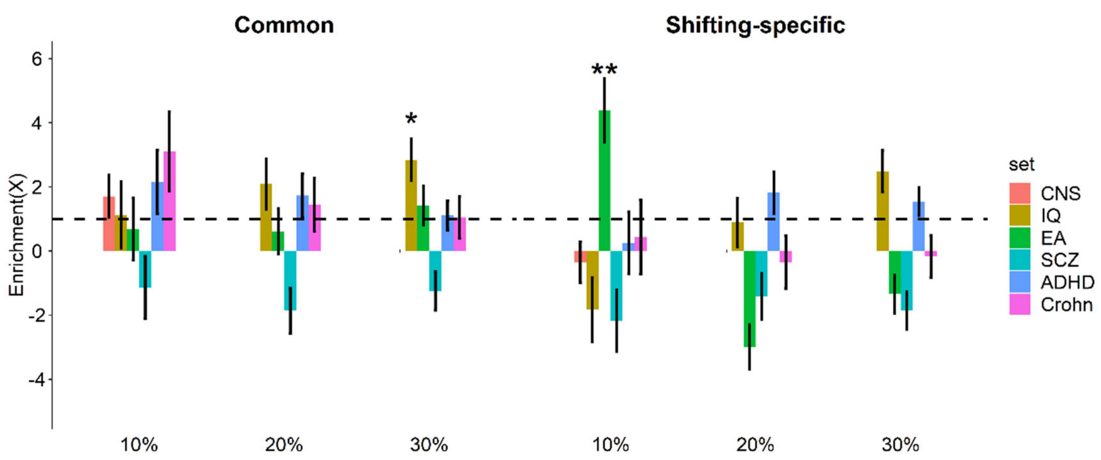

Supplementary Fig. 3. Enrichment heritability pattern of the candidate gene sets for the common EF and shifting-specific components (n = 1454 subjects). The null hypothesis of 1.0 enrichment is shown by a dashed dark horizontal line. The *p* values indicate the significance of the difference from the expectation. Error bars represent the SE of enrichment folds (= SE(set) /%SNP). Significant results after FDR-BH correction are marked with asterisks (** represents *p* values < .01, * represents *p* values < .05, exact *p* values are provided in Source Data file, one-sided test). We selected the top 10% SNPs of the ranked genome data as trait-associated variants. CNS: genes preferentially expressed in the central nervous system; IQ: SNPs associated with human intelligence; EA: SNPs associated with educational attainment; SCZ: schizophrenia-associated SNPs; ADHD: ADHD-associated SNPs; Crohn: SNPs associated with Crohn's disease. Please note that the heritability (h^2) is per definition non-negative. However, in some cases, negative values can be obtained from unbiased estimators. As discussed in Yang et al.[8] and Elhezzani[9], a negative estimate could be due to a close-to-zero true value or substantial uncertainty in estimation. For this reason, negative enrichment was thus not considered further. Source data are provided as a Source Data file.

Supplementary Table 1. Descriptive statistics for the nine EF measures

	N	Mean	SD	Min	Max	Skew	Kurtosis	IC
Anti-saccade(d')	2015	.25	.13	.00	.58	.33	-.59	.90
Stop-signal (ms)	1862	215.18	40.89	110.33	320.01	.10	.01	.55[a]
Stroop (ms)	1990	138.67	82.36	-77.75	347.31	.40	.07	.33
Category (ms)	2042	295.22	138.70	-89.64	667.51	.31	-.08	.51
Color-shape (ms)	1965	326.28	196.78	-181.69	818.83	.57	.10	.70
Number-letter (ms)	1901	300.98	172.15	-153.35	729.32	.62	.04	.79
Keep track (nc)	1980	30.70	3.11	23.00	36.00	-.52	-.27	.67[b]
Spatial 2-back (d')	1940	2.80	.93	.20	4.65	-.11	-.32	.76
Letter 3-back (d')	1957	1.98	.89	-.07	4.40	.74	-.03	.81

SD = standard deviation; Min = minimum; ax = maximum. IC= internal consistency. d' = dprime; ms = millisecond; nc = number of corrected trials.

Unless otherwise noted, IC was calculated by adjusting split-half (odd–even) correlations with the Spearman-Brown prophecy formula.

[a] For the stop-signal task, we used four stair-cases with different starting values, which were randomly mixed together. We therefore calculated the SSRT for the last two blocks. The split-half correlation was calculated based on 391 subjects who had at least one inflection point for one of the four stair cases in both blocks were used.

[b] IC was calculated using Cronbach's alpha across 4 sets of trials at each difficulty level for keep track.

Supplementary Table 2. Pearson Correlation of the nine EF measures

	1	2	3	4	5	6	7	8	9
1.Anti-saccade	—								
2.Stop-signal	.26***	—							
3.Stroop	.11***	.10***	—						
4.Category	.09***	.06*	.06**	—					
5.Color-shape	.03	.05*	.05*	.24***	—				
6.Number-letter	.10***	.08**	.08***	.31***	.32***	—			
7.Keep track	.16***	.16***	.01	.01	.04	.07**	—		
8.Spatial 2-back	.22***	.16***	.07**	.07**	.06**	.10***	.14***	—	
9.Letter 3-back	.22***	.18***	.05*	.04	.07**	.07**	.22***	.31***	—

The significant results after FDR-BH correction are noted with asterisks (*** represents p values < .001, * represents p values < .05, exact p values are provided in Source Data file, two-sided test). Source data are provided as a Source Data file.

Supplementary Table 3. Model fit statistics of the 12 EF latent variable models (list-wise deletion)

Model	χ^2	df	CFI	RMSEA	SRMR
**Correlated-factors models**					
**1. I+U+S**	**44.73**	**24**	**.98**	**.02**	**.02**
2. S/I+U	270.89	26	.73	.08	.07
**3. U/I+S**	**84.44**	**26**	**.93**	**.04**	**.03**
4. S/U+I	347.16	26	.64	.09	.07
5. G	380.25	27	.60	.10	.07
**Bifactor models**					
6. C+I+U+S	28.91	18	.99	.02	.02
**7. C+I+S**	**46.50**	**21**	**.97**	**.03**	**.03**
8. C+I+U	158.44	21	.85	.07	.06
**9. C+U+S**	**29.44**	**21**	**.99**	**.02**	**.02**
10. C+I	346.93	24	.64	.10	.07
**11. C+S**	**82.45**	**24**	**.93**	**.04**	**.03**
12. C+U	259.38	24	.74	.08	.07

The good-fit models are indicated in bold. It is worth noting that although the fit indices of the bifactor three-factor (C+I+U+S) model were good (CFI= .99, RMSEA = .02, SRMR= .02), the three tasks' loadings on inhibiting-specific component (anti-saccade: p = .55, stop-signal: p = .39, Stroop: p = .43) and updating-specific component (keep track: p = .20, spatial 2-back: p = .20, letter 3-back: p = .10) were not significant.

Supplementary Table 4. CPM prediction results using two other thresholds

Model	component	*p = .01*		*p = .1*	
		r	$p_{(FDR\text{-}BH)}$	r	$p_{(FDR\text{-}BH)}$
I+U+S	I	.15	6.5e-4	.18	2.17e-4
	U	.15	5.2e-4	.19	2.17e-4
	S	.07	.10	.06	.09
U/I+S	U/I	.16	4.3e-4	.19	2.17e-4
	S	.07	.10	.06	.09
C+I+S	C	.16	4.3e-4	.19	2.17e-4
	I	.06	.11	.06	.09
	S	.10	.03	.08	.06
C+U+S	C	.16	5.2e-4	.19	2.17e-4
	U	-.01	.57	.07	.19
	S	.09	.04	.08	.06
C+S	C	.16	4.3e-4	.19	2.17e-4
	S	.10	.03	.08	.06

I = inhibiting or inhibiting-specific; U=updating or updating-specific; S = shifting or shifting-specific; U/I = (updating = inhibiting); C = common. Correction for multiple comparisons was performed with the FDR-BH; one-sided permutation test.

Supplementary Table 5. The specific brain regions for Common and Shifting-specific components from the CPM results

Cluster	MNI			N
	X	Y	Z	
Common component				
precentral gyrus	44	-8	57	30
inferior temporal gyrus	-56	-45	-24	29
frontal pole	8	41	-24	27
LOC	-39	-75	44	19
LOC	-41	-75	26	16
frontal pole	6	64	22	16
inferior temporal gyrus	55	-31	-17	15
MFG	48	25	27	15
frontal pole	-39	51	17	15
middle temporal gyrus	56	-46	11	15
Shifting-specific component				
LOC	37	-65	40	20
paracingulate gyrus	6	54	16	19
planum temporale	58	-16	7	16
paracingulate/SMC	7	8	51	15
planum temporale	32	-26	13	14
frontal orbital cortex	27	16	-17	14
LOC	37	-81	1	14
postcentral gyrus	42	-20	55	11
precentral gyrus	2	-28	60	11
central opercular cortex	-55	-9	12	11

SMC = supplementary motor cortex, MFG = middle frontal gyrus, LOC= lateral occipital cortex. N = the number of contributing edges each node had.

Supplementary Table 6. The brain regions for Common and Shifting-specific components from the conjunction of CPM and Neurosynth results

Components	Neurosynth Cluster	No. of Voxels	COG			Node Atlas
			X	Y	Z	
Common	MFG	50	48	25	27	MFG (86 voxels, MNI= 48,25,27)
Shifting-specific	paracingulate/ SMC	45	6	9	51	Paracingulate (54 voxels,MNI=7,8,51)
	LOC	27	34	-64	40	LOC (72 voxels, MNI=37, -65,40)

SMC = supplementary motor cortex, MFG = middle frontal gyrus, LOC= lateral occipital cortex, COG = center of gravity.

Supplementary Table 7. Enrichment patterns of the Allen brain expression candidate gene sets (using other gene boundaries)

Components	Gene sets	Gene boundary	$P_{(95\%)}$	$P_{(75\%)}$	EXP#genes (75%)	OBS#genes (75%)	$P_{(FDR\text{-}BH)}$ (75%)
Common	MFG	25kb	.17	3.0e-4	244	287	3.6e-3
		50kb	.15	9.4e-3	239	266	5.6e-2
	SCG	25kb	.38	.10	241	257	.14
		50kb	.33	.09	237	252	.22
	LOC	25kb	.37	.09	245	262	.14
		50kb	.31	.46	240	239	.50
	BG	25kb	.69	.12	242	256	.14
		50kb	.11	.09	238	253	.22
Updating-specific	MFG	25kb	.14	.08	243	261	.14
		50kb	.57	.16	241	252	.27
	SCG	25kb	.16	.11	242	257	.14
		50kb	.60	.25	238	245	.38
	LOC	25kb	.14	.14	243	256	.15
		50kb	.74	.11	240	254	.22
	BG	25kb	.04	.43	242	244	.43
		50kb	.82	.68	239	232	.68
Shifting-specific	MFG	25kb	.29	.04	145	162	.14
		50kb	.32	.05	143	158	.20
	SCG	25kb	.17	.02	139	161	.12
		50kb	.64	6.8e-3	138	161	5.6e-2
	LOC	25kb	.30	.09	150	163	.14
		50kb	.49	.33	148	152	.44
	BG	25kb	.96	.12	139	151	.14
		50kb	.89	.39	138	140	.47

See Table 3 for definition of acronyms. Correction for multiple comparisons was performed with the FDR-BH. One-sided permutation test.

Supplementary Table 8. High dimensional mediation analyses results

Components	Gene boundary	VIE	VDE	PVM	$p_{permutation}$	$p_{(FDR-BH)}$
Common	25kb	.023	.093	.201	4.4e-3	9.4e-3
	35kb	.027	.114	.190	4.7e-3	9.4e-3
	50kb	.034	.142	.193	4.2e-3	9.4e-3
Shifting-specific	25kb	.016	-.096	-.203	.995	.996
	35kb	.016	-.104	-.176	.996	.996
	50kb	.019	-.106	-.220	.996	.996

Exposure: genotype of MFG-related genes or SCG-related genes; Mediator: selected edges in CPM for EF components; Outcome: EF components. VIE = variance indirect effect, VDE = variance direct effect, PVM = proportion of the variance mediated. Correction for multiple comparisons was performed with the FDR-BH. One-sided permutation test.

Supplementary References

1. Chen, J., Bardes, E.E., Aronow, B.J. & Jegga, A.G. ToppGene Suite for gene list enrichment analysis and candidate gene prioritization. *Nucleic Acids Res* **37**, W305-11 (2009).
2. Harris MA, C.J., Ireland A, Lomax J, Ashburner M, Foulger R, Eilbeck *et al.* The Gene Ontology (GO) database and informatics resource. *Nucleic Acids Res* **32**(2004).
3. Kanehisa M, G.S., Hattori M, Aoki-Kinoshita KF, Itoh M, & Kawashima S, K.T., Araki M, Hirakawa M. From genomics to chemical genomics: new developments in KEGG. *Nucleic Acids Res* **34**(2006).
4. Karp PD, O.C., Moore-Kochlacs C, Goldovsky L, Kaipa P, & Ahren D, T.S., Darzentas N, Kunin V, Lopez-Bigas N. Expansion of the BioCyc collection of pathway/genome databases to 160 genomes. *Nucleic Acids Res* **33**, 6083-6089 (2005).
5. Joshi-Tope G, G.M., Vastrik I, D'Eustachio P, Schmidt E, de, Bono B, J.B., Gopinath GR, Wu GR, Matthews L, Lewis S, Birney & E, S.L. Reactome: a knowledgebase of biological pathways. *Nucleic Acids Res* **33**(2005).
6. Dahlquist KD, S.N., Vranizan K, Lawlor SC, Conklin BR. GenMAPP, a new tool for viewing and analyzing microarray data on biological pathways. *Nat Genet* **31**, 19-20 (2002).

7. Benjamini, Y. & Hochberg, Y. controling the false discovery rate a practical and powerful approach to multiple testing. *JOURNAL OF THE ROYAL STATISTICAL SOCIETY.Series B(Methodological)* **57**, 289-300 (1995).
8. Yang, J. *et al.* Ubiquitous polygenicity of human complex traits: genome-wide analysis of 49 traits in Koreans. *PLoS Genet* **9**, e1003355 (2013).
9. Elhezzani, N.S. Improved estimation of SNP heritability using Bayesian multiple-phenotype models. *Eur J Hum Genet* **26**, 723-734 (2018).
