# Supplementary material for: A cognitive neurogenetic approach to uncovering the structure of executive functions
Source: Nat Commun. 2022 Aug 6;13:4588. doi: 10.1038/s41467-022-32383-0 (PMC9357028; doi:10.1038/s41467-022-32383-0)
Supplement: Supplementary file 3 — Description of Additional Supplementary Files [file 41467_2022_32383_MOESM3_ESM.pdf]

File name: Supplementary Data 1

Description: The Supplementary Data contains the detailed lists of genes with enhanced expression in the brain regions associated with the common (MFG), shifting-specific (SCG, LOC), and updating-specific (BG) components according to the AHBA; the functional enrichment of the MFG- and SCG-related genes using ToppGene suite; the behavioral data of the 9 tasks that is used to construct the EFs models.
