# Supplementary material for: A cognitive neurogenetic approach to uncovering the structure of executive functions
Source: Nat Commun. 2022 Aug 6;13:4588. doi: 10.1038/s41467-022-32383-0 (PMC9357028; doi:10.1038/s41467-022-32383-0)
Supplement: Supplementary file 5 — Reporting Summary [file 41467_2022_32383_MOESM5_ESM.pdf]

Corresponding author(s): Gui Xue

Last updated by author(s): Jul 4, 2022

Reporting Summary

Nature Portfolio wishes to improve the reproducibility of the work that we publish. This form provides structure for consistency and transparency in reporting. For further information on Nature Portfolio policies, see our Editorial Policies and the Editorial Policy Checklist.

Statistics

For all statistical analyses, confirm that the following items are present in the figure legend, table legend, main text, or Methods section.

n/a	Confirmed	
☐	☒	The exact sample size (*n*) for each experimental group/condition, given as a discrete number and unit of measurement
☐	☒	A statement on whether measurements were taken from distinct samples or whether the same sample was measured repeatedly
☐	☒	The statistical test(s) used AND whether they are one- or two-sided<br>*Only common tests should be described solely by name; describe more complex techniques in the Methods section.*
☐	☒	A description of all covariates tested
☐	☒	A description of any assumptions or corrections, such as tests of normality and adjustment for multiple comparisons
☐	☒	A full description of the statistical parameters including central tendency (e.g. means) or other basic estimates (e.g. regression coefficient) AND variation (e.g. standard deviation) or associated estimates of uncertainty (e.g. confidence intervals)
☐	☒	For null hypothesis testing, the test statistic (e.g. *F*, *t*, *r*) with confidence intervals, effect sizes, degrees of freedom and *P* value noted<br>*Give P values as exact values whenever suitable.*
☒	☐	For Bayesian analysis, information on the choice of priors and Markov chain Monte Carlo settings
☒	☐	For hierarchical and complex designs, identification of the appropriate level for tests and full reporting of outcomes
☐	☒	Estimates of effect sizes (e.g. Cohen's *d*, Pearson's *r*), indicating how they were calculated

Our web collection on statistics for biologists contains articles on many of the points above.

Software and code

Policy information about availability of computer code

Data collection	MATLAB R2013a was used to collect behavioral data of the nine executive functions.<br>3.0 T Siemens MRI Trio scanner was used to collect neuroimaging data.
Data analysis	MATLAB R2021b was used to preprocess and calculate the indices of the nine executive functions.<br>“lavaan” package (version 0.6-9) in R software (version 4.1) was used to estimate the latent variable models of EFs.<br>Gretna v2.0 and AFNI 3dTproject (AFNI program: 3dTproject (nih.gov)) was used to perform resting-state fMRI preprocessing.<br>Plink1.9 was used to preprocess genome data.<br>GCTA V1.26 was used to estimate the heritability of EF components and their genetic correlation.<br>Preprocessing of the Allen Human Brain Atlas was done using abagen (https://github.com/netneurolab/abagen).<br>MAGENTA was used to perform gene set enrichment analysis.<br>ToppGene suite (https://toppgene.cchmc.org/) was used to perform functional gene set enrichment analysis of the candidate genes.

For manuscripts utilizing custom algorithms or software that are central to the research but not yet described in published literature, software must be made available to editors and reviewers. We strongly encourage code deposition in a community repository (e.g. GitHub). See the Nature Portfolio guidelines for submitting code & software for further information.

Data

Policy information about availability of data

All manuscripts must include a data availability statement. This statement should provide the following information, where applicable:

- Accession codes, unique identifiers, or web links for publicly available datasets
- A description of any restrictions on data availability
- For clinical datasets or third party data, please ensure that the statement adheres to our policy

The AHBA is available at https://human.brain-map.org/static/download/.
The Neurosynth database is available at https://www.neurosynth.org/analyses/terms/
The sources of the GWAS summary results are as follows: schizophrenia and ADHD (https://www.med.unc.edu/pgc/download-results/); intelligence (https://ctg.cncr.nl/software/summary_statistics/); educational attainment (https://www.thessgac.org/data); and Crohn's disease (https://www.ibdgenetics.org/downloads.html).
The list of genes preferentially expressed in the central nervous system was obtained from the corresponding author of the study (Lee et al., 2012).
UCSC hg19 assembly (UCSC Human Gene Sorter).
Behavioral data to estimate EFs models can be found in Supplementary Data. Raw data of the Cognitive Neurogenetic Study of Han Chinese Young Adults (CNSCYA) Project are available from the corresponding author on reasonable request. Restriction of raw data is to protect the privacy of participants. Source data are provided with this paper.

Field-specific reporting

Please select the one below that is the best fit for your research. If you are not sure, read the appropriate sections before making your selection.

☒ Life sciences ☐ Behavioural & social sciences ☐ Ecological, evolutionary & environmental sciences

For a reference copy of the document with all sections, see nature.com/documents/nr-reporting-summary-flat.pdf

Life sciences study design

All studies must disclose on these points even when the disclosure is negative.

Sample size	No statistical methods were used to pre-determine sample sizes. The sample size consists of all individuals that remain after quality control of the data. Detailed information on the samples used, as well as the exclusion/inclusion criteria, are provided in the Methods.
Data exclusions	Subjects were excluded due to poor task performance (40~194 subjects for the nine tasks), non-Han Chinese (9 subjects) or close genomic relationships (3 subjects), or poor-quality resting-state data (a translation greater than 3 mm in any direction, or a rotation greater than 3 degrees, or lacking whole-brain coverage) (91 subjects), or missing gender or age information (4 subjects).
Replication	The results can be replicated by following the methods described in the manuscript and running the codes available in our GitHub repository. The data from the ABHA, Neurosynth, summary statistic results of GWAS are open access. Behavioral data to estimate EFs models can be found in Supplementary Data. Brain and gene Data from the CNSCYA project require additional data requests to access restricted in-house datasets for replication of findings.
Randomization	All know technical and biological covariates were included in the statistical models.
Blinding	Blinding was not relevant to our study for all participants in the current study were healthy adults.

Reporting for specific materials, systems and methods

We require information from authors about some types of materials, experimental systems and methods used in many studies. Here, indicate whether each material, system or method listed is relevant to your study. If you are not sure if a list item applies to your research, read the appropriate section before selecting a response.

Materials & experimental systems

n/a	Involved in the study	
☒	☐	Antibodies
☒	☐	Eukaryotic cell lines
☒	☐	Palaeontology and archaeology
☒	☐	Animals and other organisms
☐	☒	Human research participants
☒	☐	Clinical data
☒	☐	Dual use research of concern

Methods

n/a	Involved in the study	
☒	☐	ChIP-seq
☒	☐	Flow cytometry
☐	☒	MRI-based neuroimaging

Human research participants

Policy information about studies involving human research participants

Population characteristics	Participants were of Chinese Han ancestry, healthy young adults(17~31 years). Age and gender were included as covariates or most analyses. The population characteristics is available in the Methods.
Recruitment	Participants were recruited by posting posters on campus.
Ethics oversight	This study was approved by the Institutional Review Boards (IRBs) of Beijing Normal University and Southwest University, China.

Note that full information on the approval of the study protocol must also be provided in the manuscript.

Magnetic resonance imaging

Experimental design

Design type	resting-state fMRI
Design specifications	~6 minutes per subjects,
Behavioral performance measures	Not relevant

Acquisition

Imaging type(s)	resting-state functional MRI and structural MRI
Field strength	3T
Sequence & imaging parameters	Anatomical MRI scans were acquired using a T1-weighted, three-dimensional, gradient-echo pulse sequence. Parameters for this sequence were as follows: Repetition time/echo time/θ = 2,530 ms/3.39 ms/7degree, field of view = 256 × 256 mm, matrix = 192 × 256, and slice thickness = 1.33 mm, 144 sagittal slices for the Beijing data. For Chongqing sample, repetition time/echo time/θ = 2600 msec/3.02msec/8degree, field of view = 256 × 256 mm, matrix = 256 × 256, and slice thickness = 1.00 mm. A total of 176 sagittal slices were acquired. Functional scanning used a gradient echo EPI sequence with PACE (prospective acquisition correction). The following parameters were used: TR = 2,000 ms; TE = 30 ms; flip angle = 90degree; FOV = 200 × 200 mm2; 64 × 64 matrix size with a resolution of 3.1 × 3.1 mm2. Thirty-three 3.5 mm transverse slices were used for the Beijing data. The Chongqing sample used thirty-two 3.0 mm transverse slices to cover the whole cerebrum and most of the cerebellum, TR= 2000 ms; TE = 30ms; flip angle = 90degree; FOV = 220 × 220 mm2; 64 ×64 matrix size with aresolution of 3.4 × 3.4 mm2. 200 volumes for the Beijing sample, and 242 volumes for the Chongqing sample.
Area of acquisition	whole brain

Diffusion MRI ☐ Used ☒ Not used

Preprocessing

Preprocessing software	Gretna v2.0 and AFNI softwares
Normalization	warping individual functional images to the standard MNI space by applying the transformation matrix that can be derived from registering the final Template file generated by DARTEL.
Normalization template	MNI305
Noise and artifact removal	Based on the methods from a recent study (Lindquist, Geuter,Wager, & Caffo, 2019), we combined nuisance covariates,linear trends, and temporal filters (0.01–0.1 Hz) into a single regression model to avoid reinjection of the noise signals. The nuisance covariates included the average signal from the cerebrospinal fluid and white matter, the global signal, and 24 motion parameters (Friston, Williams, Howard, Frackowiak, & Robert, 1996).
Volume censoring	No volume censoring was performed.

Statistical modeling & inference

Model type and settings	a modified connectome-based predictive modeling (CPM) protocol
Effect(s) tested	The association between brain functional connectivity pattern and the EF latent components, as described in the paper.

Specify type of analysis: ☒ Whole brain ☐ ROI-based ☐ Both

Statistic type for inference
(See Eklund et al. 2016)

a nonparametric permutation test

Correction

The paper includes various different analyses and the multiple testing approach for each is described explicitly in the methods, both FDR-BH(Benjamini & Hochberg, 1995).

Models & analysis

n/a	Involved in the study
☒	☐ Functional and/or effective connectivity
☒	☐ Graph analysis
☐	☒ Multivariate modeling or predictive analysis

Multivariate modeling and predictive analysis

We applied a modified connectome-based predictive modeling (CPM) protocol (Shen et al., 2017)to explore the neural mechanism of each EF component. In particular, we used ten-fold instead of leave-one-out cross-validation in consideration of our large sample size (n=870).
